# In vitro characterization of the splicing efficiency and fidelity of the RmInt1 group II intron as a means of controlling the dispersion of its host mobile element

**DOI:** 10.1261/rna.047407.114

**Published:** 2014-12

**Authors:** Isabel Chillón, María Dolores Molina-Sánchez, Olga Fedorova, Fernando Manuel García-Rodríguez, Francisco Martínez-Abarca, Nicolás Toro

**Affiliations:** 1 Grupo de Ecología Genética, Estación Experimental del Zaidín, Consejo Superior de Investigaciones Científicas, 18008 Granada, Spain; 2 Howard Hughes Medical Institute, Chevy Chase, Maryland 20815, USA; 3 Department of Molecular, Cellular and Developmental Biology, Yale University, New Haven, Connecticut 06520, USA

**Keywords:** RNA circles, RmInt1, group II intron, ribozyme, splicing

## Abstract

Group II introns are catalytic RNAs that are excised from their precursors in a protein-dependent manner in vivo. Certain group II introns can also react in a protein-independent manner under nonphysiological conditions in vitro. The efficiency and fidelity of the splicing reaction is crucial, to guarantee the correct formation and expression of the protein-coding mRNA. RmInt1 is an efficient mobile intron found within the IS*Rm2011-2* insertion sequence in the symbiotic bacterium *Sinorhizobium meliloti*. The RmInt1 intron self-splices in vitro, but this reaction generates side products due to a predicted cryptic IBS1* sequence within the 3′ exon. We engineered an RmInt1 intron lacking the cryptic IBS1* sequence, which improved the fidelity of the splicing reaction. However, atypical circular forms of similar electrophoretic mobility to the lariat intron were nevertheless observed. We analyzed a run of four cytidine residues at the 3′ splice site potentially responsible for a lack of fidelity at this site leading to the formation of circular intron forms. We showed that mutations of residues base-pairing in the tertiary EBS3–IBS3 interaction increased the efficiency and fidelity of the splicing reaction. Our results indicate that RmInt1 has developed strategies for decreasing its splicing efficiency and fidelity. RmInt1 makes use of unproductive splicing reactions to limit the transposition of the insertion sequence into which it inserts itself in its natural context, thereby preventing potentially harmful dispersion of IS*Rm2011-2* throughout the genome of its host.

## INTRODUCTION

**FIGURE 1.**
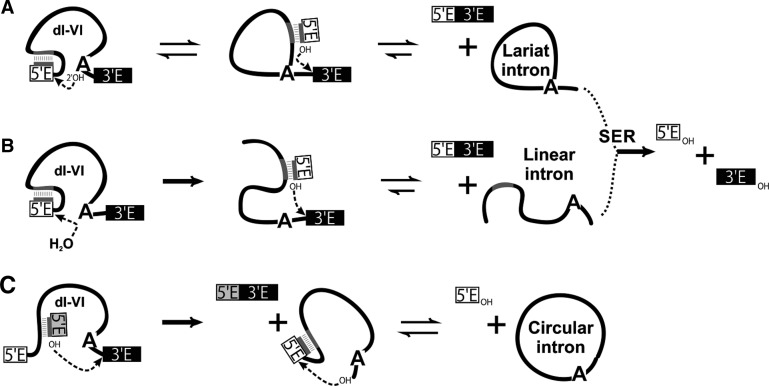
Mechanisms of group II intron excision. Schematic representations of the branching (*A*), hydrolytic (*B*), and circle formation (*C*) pathways are shown yielding lariat, linear, and circular intron, respectively. The different mechanisms are described in the text. The intron is indicated by a black line, while the exon sequences are represented as white/gray (5′ exon) or black (3′ exon) boxes. The bulged adenosine in the intron domain VI is represented as A. Dotted lines indicate the nucleophilic attack during each step of reaction.

Group II introns are catalytic RNAs that are excised from their precursor RNA in a protein-dependent manner in vivo (Pyle and Lambowitz 2006; Lambowitz and Zimmerly 2010). Self-splicing can occur in the absence of proteins in vitro, if these introns are incubated under nonphysiological conditions (Pyle 2010). As in spliceosomal introns, the ribozyme of group II introns excises the intron as a branched, lariat structure, through two sequential transesterification reactions (Fig. 1A). In the first step, the 2′ OH group of a bulged adenosine in domain 6 acts as the nucleophile for an attack on the 5′ splice site (5′ SS), producing an intron lariat-3′ exon intermediate. In the second step, the 3′ OH of the cleaved 5′ exon, which remains tightly bound to the intron via EBS1–IBS1 and EBS2–IBS2 (exon and intron binding sites 1 and 2, respectively) base-pairing interactions, acts as the nucleophile for an attack on the 3′ splice site (3′ SS), resulting in exon ligation and the excision of an intron lariat RNA (van der Veen et al. 1986; Pyle and Lambowitz 2006; Toro et al. 2007). A hydrolytic pathway, in which a water molecule acts as the nucleophile during the first step of splicing, can also occur, producing ligated exons and a linear intron (Fig. 1B; Jarrell et al. 1988; Daniels et al. 1996; Fedorova and Zingler 2007). A third pathway involves the formation of intron circles, in which the 5′ and 3′ extremities of the intron are covalently linked by a putative 5′–2′ bond (Fig. 1C; Murray et al. 2001). This alternative splicing pathway has been described both in vitro (Murray et al. 2001; Nagy et al. 2013) and in vivo (Molina-Sanchez et al. 2006, 2011). It requires the presence of a 5′ exon–intron intermediate formed by *trans*-splicing, the prior release of the 3′exon and the successive attack of the 2′ OH group of the 3′ end of the intron to the 5′ SS. In vitro, group II introns can catalyze spliced-exon reopening (SER), a reaction involving the hydrolysis of ligated exons to generate free 5′ and 3′ exons (Jarrell et al. 1988). Regardless of the splicing pathway, the free spliced intron can reinsert itself in vivo into target loci within the genome of the host organisms and can be reverse-transcribed by the intron-encoded reverse transcriptase during retrohoming (Cousineau et al. 2000; Muñoz-Adelantado et al. 2003; Zhong and Lambowitz 2003; Martinez-Abarca et al. 2004).

**FIGURE 2.**
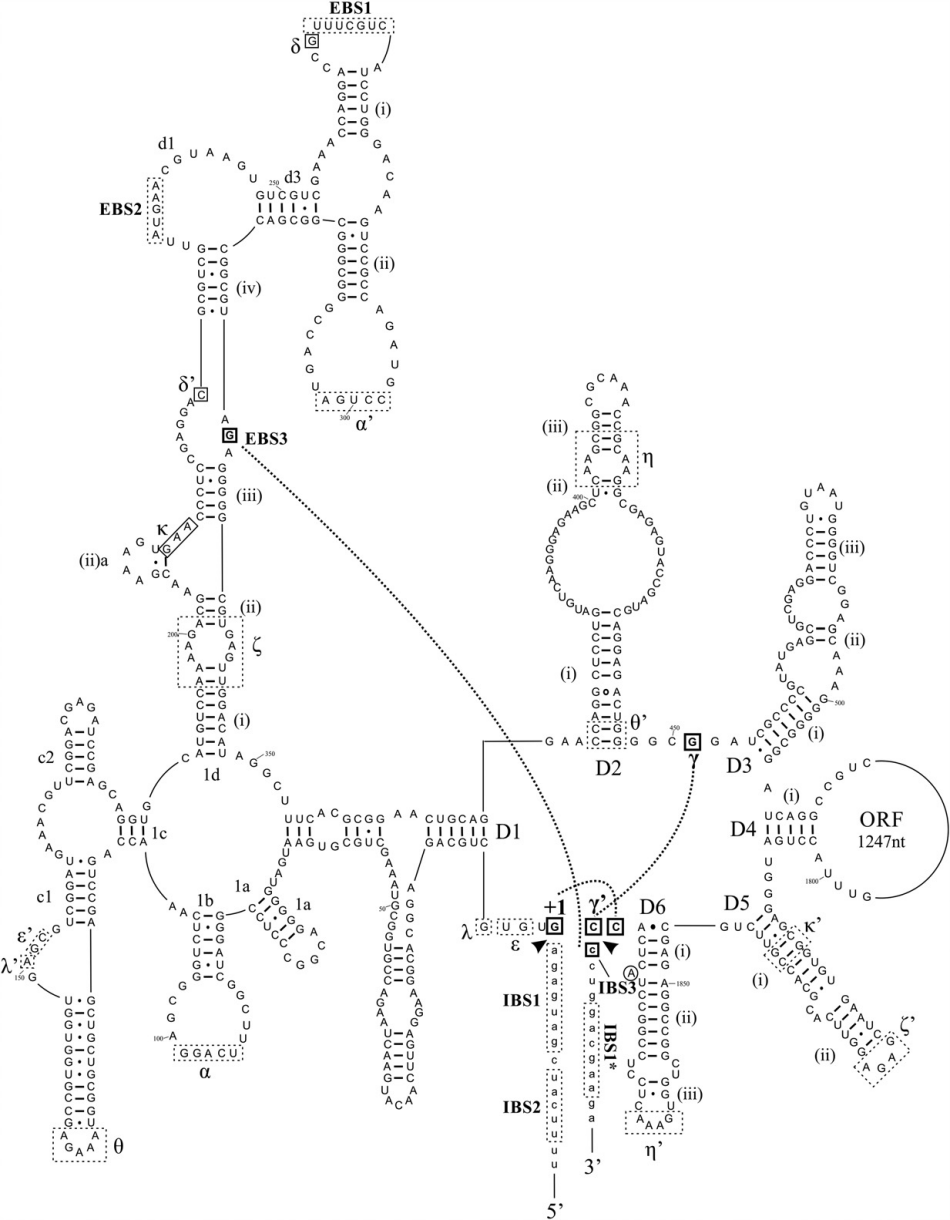
Diagram of the secondary structure of the RmInt1 group II intron. The nucleotides at the 3′ splice site (3′ SS) and the nucleotides with which they have been reported to interact are highlighted in bold letters and enclosed in boxes with solid lines. Long-range interactions between these nucleotides are denoted by dashed bold lines. Sequence elements involved in other long-range interactions are indicated by dashed boxes. Arrowheads indicate the 5′ and 3′ SS, respectively. Uppercase letters correspond to intron sequences, whereas lowercase characters correspond to exon sequences.

RmInt1 is a mobile bacterial group II intron found within the IS*Rm2011-2* insertion sequence in *Sinorhizobium meliloti*, the nitrogen-fixing symbiont of alfalfa (*Medicago sativa*) (Martinez-Abarca et al. 1998). It is a bacterial IIB3/class D intron and has a class IIB-like RNA structure, with some IIA features (Fig. 2; Zimmerly et al. 2001; Toro et al. 2003). RmInt1 self-splices in vitro through the branching and hydrolytic pathways (Costa et al. 2006a). However, the second step in splicing is particularly inefficient, resulting in the accumulation of a lariat/3′ exon intermediate and poor exon ligation. RmInt1 splicing also generates aberrant products, demonstrating a poor splicing fidelity for this intron. For instance, an unexpected truncated lariat/3′ exon intermediate, with only 10 or 11 nt in its 3′ exon, has been detected (Costa et al. 2006a). It has been suggested that this aberrant product is generated by ribozyme-promoted cleavage 3′ to the GACGAA or GACGAAG sequence (hereafter designated IBS1*) in the 3′ exon, which better matches the EBS1 (UUUCGUC) sequence than the authentic IBS1 (GAUGAGA) sequence in the 5′ exon (Costa et al. 2006b). We found that in vivo RmInt1 splicing led to the accumulation of lariat forms but that this process was inefficient, shown by a low detection of ligated exons (Chillón et al. 2011). Thus, RmInt1 functions more like a retroelement than spliceosomal intron preventing the spread of IS*Rm2011-2* in the *S. meliloti* genome.

In group II introns, certain nucleotides are conserved at intron boundaries. The consensus sequence at the 5′ end of the intron is GUGYG, whereas the consensus sequence at the 3′ end is AY (where Y is C or U). Some variations have been reported, associated with the various specific RNA subgroups (Dai et al. 2008; Candales et al. 2012). Remarkably, the RmInt1 intron shows a deviation from the consensus sequence at its 3′ end (CC) (Fig. 2). The nucleotide at the 3′ end is engaged in a Watson–Crick interaction with a purine located in the joining segment separating domain 2 from domain 3 (J2/3) (γ–γ′ interaction) (Jacquier and Michel 1990). The penultimate nucleotide is predicted to be involved in a non-Watson–Crick interaction with the first nucleotide of the intron, occurring after lariat formation (Chanfreau and Jacquier 1993). The presence of a pyrimidine at the penultimate position of RmInt1 differentiates this intron from all other group II introns in terms of consensus sequences at the 3′ end (AY), but also from eukaryotic pre-mRNA introns (consensus sequence at the 3′ end: AG), which are thought to be the phylogenetic descendants of group II introns (Sharp 1991; Fica et al. 2013). In addition, the CC dinucleotide at the 3′ end of RmInt1 is followed by another pair of cytidine residues at the 5′ end of the 3′ exon. The first C nucleotide in the 3′ exon is involved in a Watson–Crick base-pairing, as part of the EBS3–IBS3 interaction (exon and intron binding sites 3, respectively) with a guanosine residue located in the ID^(iii)^–ID^(iv)^ internal loop. Thus, the 3′ SS of RmInt1 forms a characteristic run of four cytidine residues (CCCC), potentially leading to the formation of an ambiguous 3′ SS.

We investigated the reasons for the low efficiency and fidelity of RmInt1 in vitro. We found that deletion of the predicted IBS1* resulted in a significant decrease of side products. We also analyzed the role of the run of four cytidine residues at the 3′ SS and revealed that this sequence was responsible for the formation of aberrant products (truncated circles or circles carrying extra residues) migrating with the lariat intron. These observations provide insight into the biological role of RmInt1, which requires impairment of its enzyme to limit the potentially harmful dispersion of other mobile genetic elements in the host genome.

## RESULTS

### The splicing fidelity of the RmInt1 intron is affected by an alternative EBS1–IBS1 pairing in vitro

**FIGURE 3.**
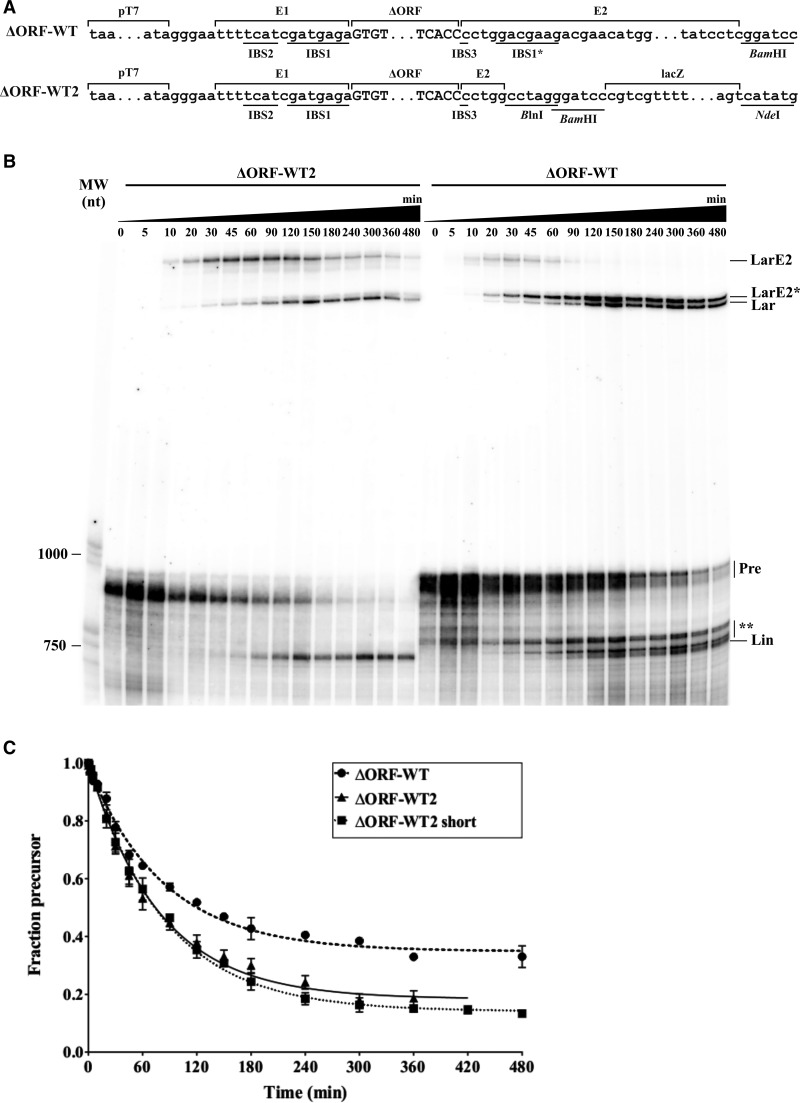
Self-splicing assay for RmInt1 ΔORF-WT and ΔORF-WT2 transcripts. (*A*) Schematic diagram of the RmInt1 WT constructs used in this study. ΔORF-WT was previously described as Δ15 or pLM1 (Costa et al. 2006a,b). (*B*) Self-splicing assay for the ΔORF-WT (*right* panel) and ΔORF-WT2 (*left* panel) transcripts. Reaction time points and nucleotide size markers are indicated at the *top* and the *left* of the gel, respectively. Possible interpretations of the various bands observed are provided on the *right*. (Lar) intron lariat; (LarE2) intron lariat-3′ intermediate; (LarE2*) intron lariat-3′ intermediate with truncation of the first 10 or 11 nt of the 3′ intron; (Pre) precursor; (Lin) intron linear, and (**) the presence of other linear side products of various lengths. (*C*) The reactivity of three RmInt1 WT transcripts is shown. Experimental data for the disappearance of precursor RNA over time were fitted with a first-order exponential equation with endpoint correction. Error bars indicate the standard error of the mean over two or three trials.

It has been suggested that a putative alternative IBS1 sequence (IBS1*) located in the 3′exon, may compete with the authentic sequence (located in D1) for the RmInt1 intron during splicing in vitro, leading to the formation of an unconventional product (Fig. 3B, right panel; Costa et al. 2006a). We investigated the effect of the IBS1* sequence on RmInt1 splicing in vitro, by deleting this sequence from the 3′ exon and analyzing the splicing rate and products (Fig. 3B).

We trimmed the 3′ exon of the ΔORF-WT RmInt1 intron construct (Costa et al. 2006a) to its first 5 nt, retaining the IBS3 sequence but not the IBS1* sequence. We then added a region corresponding to the *Escherichia coli lac*Z gene, to enlarge the trimmed 3′ exon, such that it was of similar length to ΔORF-WT (Fig. 3A). The ΔORF-WT construct and the newly engineered ΔORF-WT2 construct were then used in a self-splicing assay in vitro, with analyses carried out at various time points during the reaction (Fig. 3B). As expected, ΔORF-WT gave rise to multiple side products, in both the branching and hydrolytic pathways, and these products had previously been shown to contain an extra 10 or 11 nt from the 3′ SS (Costa et al. 2006a). In contrast, ΔORF-WT2 yielded products corresponding in size to the lariat intron or circle, lariat intron–3′ exon intermediate, and linear intron. The linear intron-3′ intermediate could not be distinguished from the intron precursor due to the short extension of the 5′ exon, although this form was taken into account for the calculation of reaction kinetics (see Materials and Methods). Interestingly, kinetic analyses showed similar apparent first-order rate constants for the conversion of precursor RNA into products for both the ΔORF-WT and ΔORF-WT2 transcripts (0.0120 ± 0.0008 and 0.0127 ± 0.0007 min^−1^, respectively). However, the degree of completion of the reaction differed significantly for the two constructs: 82% of the ΔORF-WT2 precursor reacted, versus only 65% of the ΔORF-WT precursor, suggesting a role for IBS1* in affecting the amplitude of the reaction (Fig. 3C). We also ruled out a possible effect of the *lac*Z sequence in the 3′ exon, by reacting another transcript trimmed to 5 nt but lacking the *lac*Z sequence (Fig. 3C). The rate and amplitude of the reaction were similar for this short transcript (0.0119 ± 0.0006 min^−1^ and 86%, respectively) and for ΔORF-WT2.

Thus, the IBS1* sequence located in the 3′ exon decreases the fidelity and amplitude of the RmInt1 self-splicing reaction in vitro.

### RmInt1 displays a lack of fidelity for circle formation, but that is not translated to ligated exons

**FIGURE 4.**
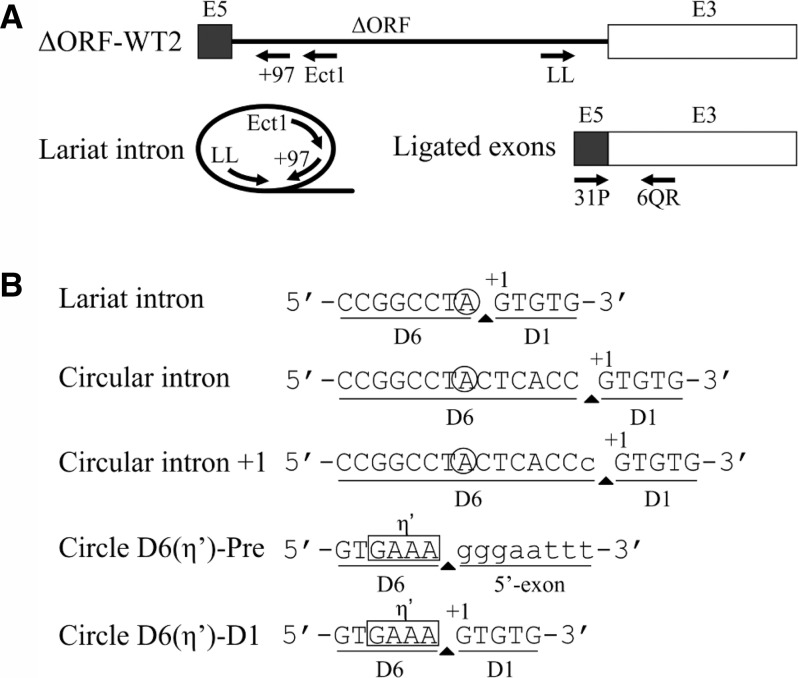
Identification of splicing forms. (*A*) Schematic diagram of the ΔORF-WT2 precursor and ligated exons with the primers (arrows) used for reverse transcription and PCR. (*B*) Sequences of the lariat and circular forms produced after splicing of the ΔORF-WT2 transcript. A circular intron, which was not observed for the wild-type ΔORF-WT2 transcript, is also shown for clarity. Uppercase letters correspond to intron sequences, whereas lowercase characters correspond to exon sequences. The covalent bond joining the two ends to form a circle is indicated by an arrowhead. The bulging A is surrounded by a circle. The first nucleotide of the intron is indicated by +1. D1 is domain 1 of the intron and D6 is domain 6 of the intron. η′ is the tetraloop involved in the tetraloop-receptor η–η′ tertiary interaction between intron domains 2 and 6.

We characterized the bands of sizes corresponding to the lariat intron or circle from the ΔORF-WT2 self-splicing reaction by gel extraction, followed by reverse transcription with the Ect1 primer and further amplification with primers LL and +97, followed by the sequencing of individual clones (Fig. 4A). Together with the expected intron lariat form, we detected circular products: an intron form with a truncated domain 6 linked either to the extremity of the 5′ exon or the beginning of the intron, and a circular intron form containing an extra residue (cytidine in position +1 with respect to the 3′ SS) (Table 3, below; Fig. 4B). We investigated the formation of correct ligated exons by examining the sequence of the splice junction, which was amplified from an unfractionated in vitro splicing reaction of ΔORF-WT2 by RT-PCR with primers binding to the 3′ and 5′ exons (Fig. 4A). All the sequences analyzed (35) corresponded to the expected exon junction.

Our sequencing data show that, along with the lariat intron, atypical circular forms were produced during the splicing reaction of the ΔORF-WT2 transcript whereas the ligated exons we analyzed were likely formed from typical splicing reactions leading to lariat formation. Nevertheless, we cannot rule out that other low abundant exonic products were formed alongside the atypical circles could be produced, and that our methods were not sensitive enough to detect them.

### Analyses of mutations at the 3′ splice site reveal an intrinsic defect of the RmInt1 intron affecting splicing fidelity at the 3′ splicing junction

**FIGURE 5.**
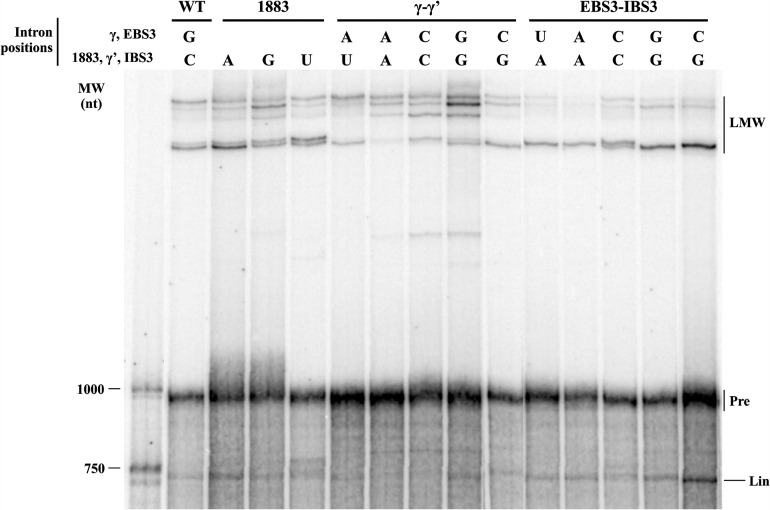
Self-splicing activity of the WT and mutant RmInt1introns at positions 329 (EBS3), 452 (γ), and 1883 and 1884 (γ′) of the intron and +1 (IBS3) of the 3′ exon. Splicing reactions were carried out for 2 h and the products were run in parallel. LMW includes all low-mobility molecules of similar electrophoretic mobility to the lariat-3′ intron intermediate and lariat intron; (Pre) the intron precursor; (Lin) the linear intron.

We hypothesized that the unusual run of four cytidines at the 3′ SS of RmInt1 might be responsible for the lack of fidelity and the formation of atypical circle molecules. We tested this hypothesis by introducing nucleotide substitutions at positions 1883 (penultimate) and 1884 (γ′) of the intron and at positions +1 (IBS3) of the 3′ exon of ΔORF-WT2 (we numbered intron positions in accordance with the full-length RmInt1 intron, although this construct lacks the ORF sequence) (Fig. 2). As these nucleotides at the 3′ SS are involved in long-range interactions with nucleotides at other positions in the intron sequence (Fig. 2; Jacquier and Michel 1990; Costa et al. 2000), we also introduced the corresponding compensatory substitutions at positions 329 (EBS3) and 452 (γ) of the intron. We analyzed the reaction rates and products (Table 1; Fig. 5), and compared them with the ligated exon rates calculated for each mutant (Table 2). We also cloned and sequenced the splicing products of selected mutants (Table 3).

**TABLE 1.**
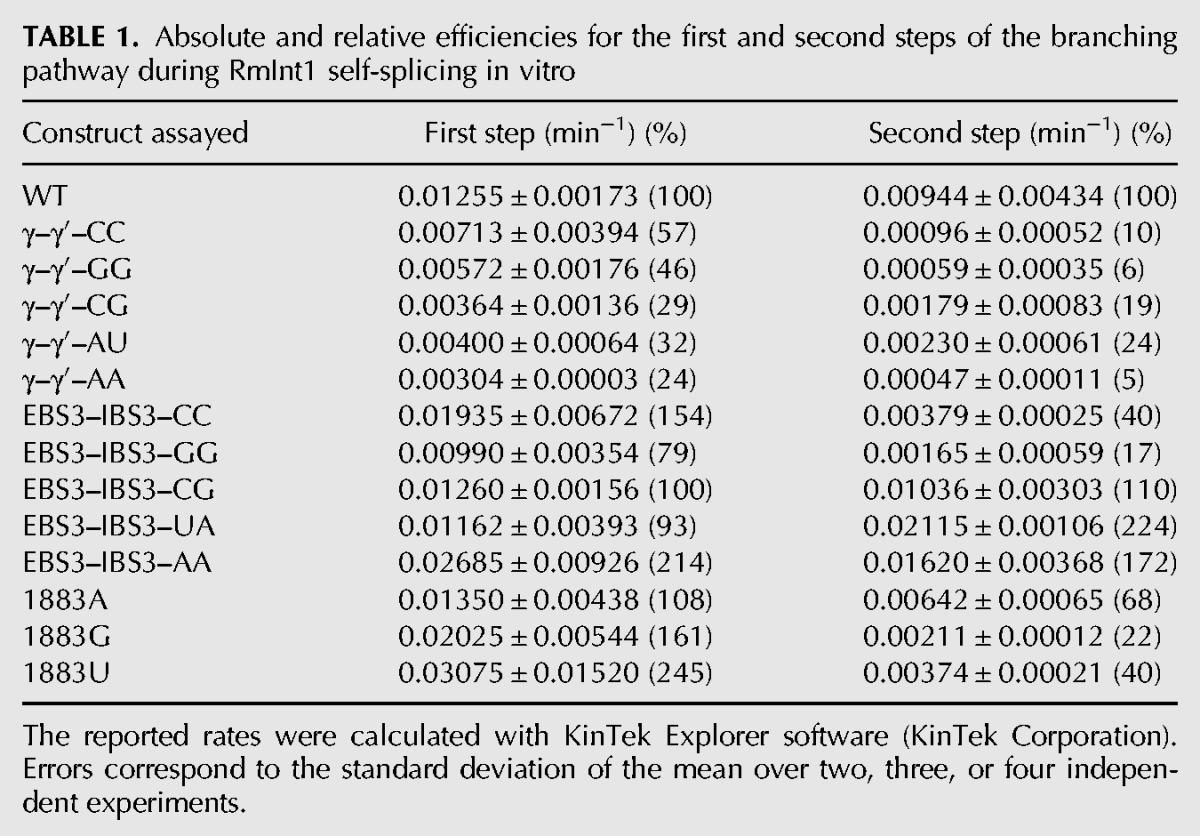
Absolute and relative efficiencies for the first and second steps of the branching pathway during RmInt1 self-splicing in vitro

**TABLE 2.**
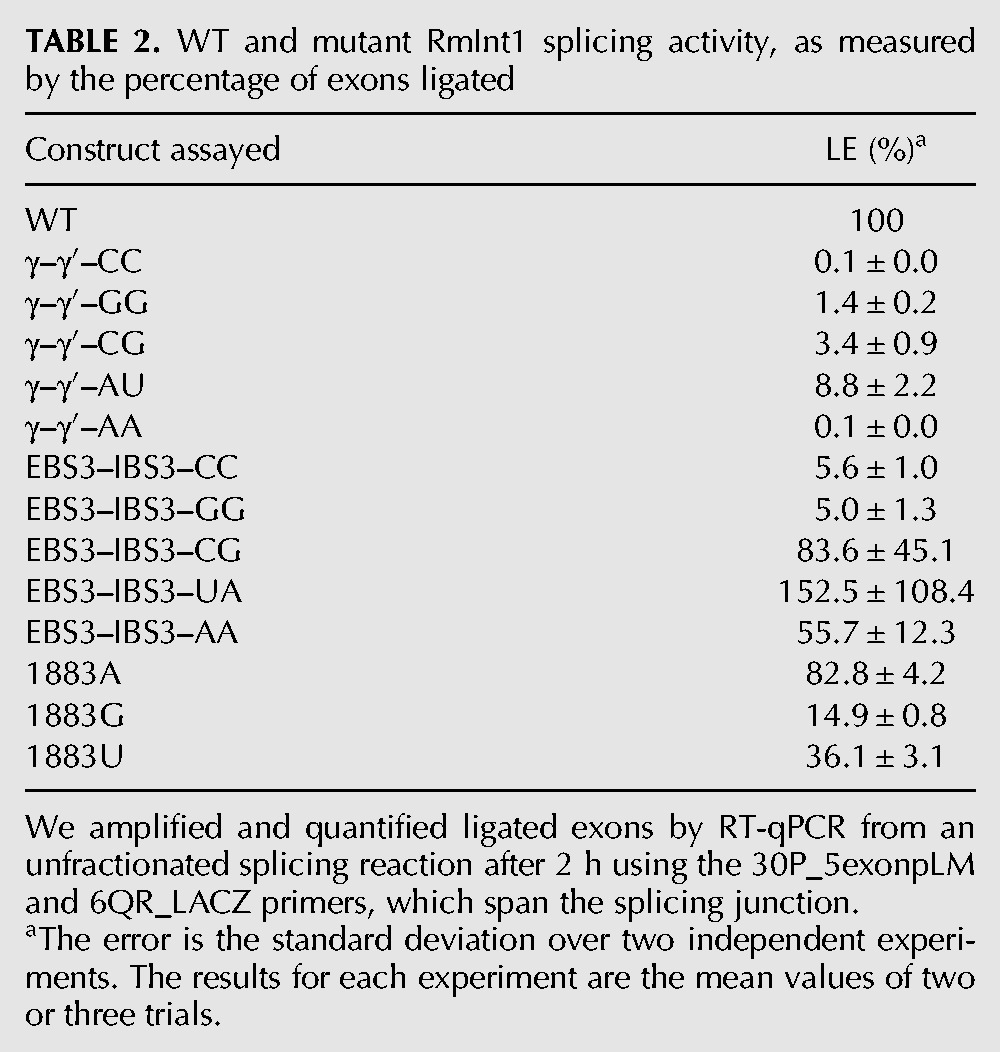
WT and mutant RmInt1 splicing activity, as measured by the percentage of exons ligated

**TABLE 3.**
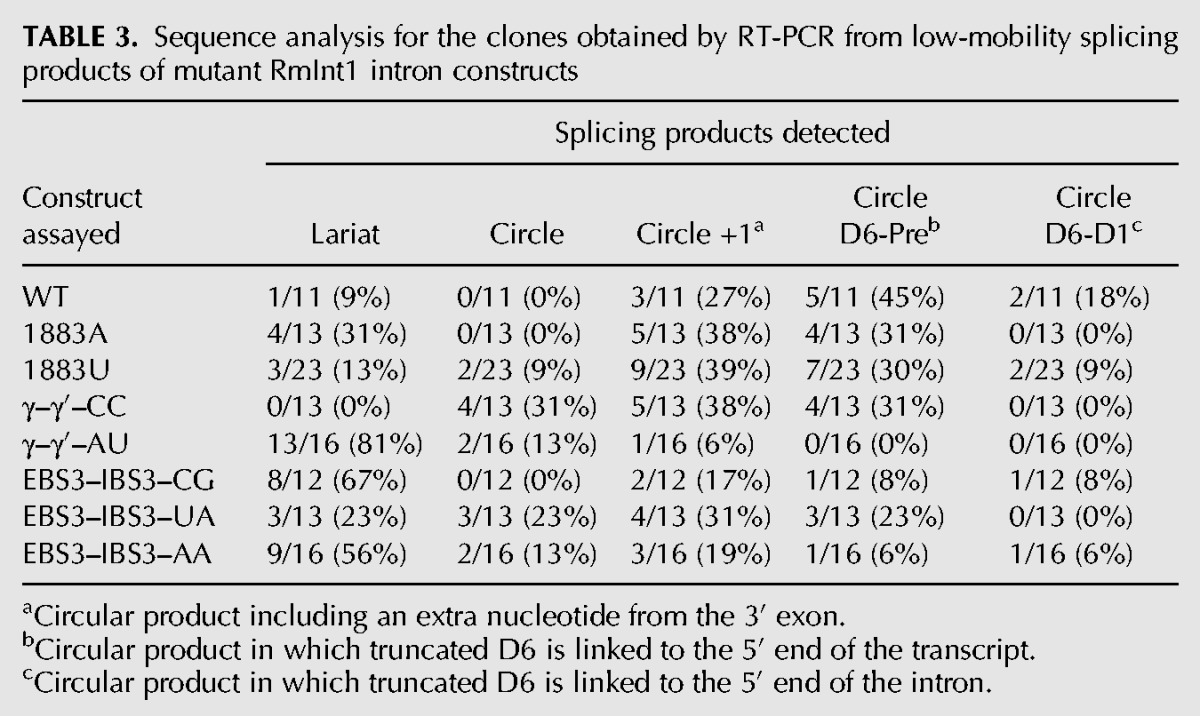
Sequence analysis for the clones obtained by RT-PCR from low-mobility splicing products of mutant RmInt1 intron constructs

Substitutions at the penultimate position of the intron (C1883) did not impair the first step of splicing, but all such substitutions resulted in defects in the second splicing reaction, with the 1883G and 1883U mutants the most severely affected (Table 1). As expected, this defect in the second splicing reaction resulted in a decrease in the rate of exon ligation (Table 2). The 1883A mutation is the substitution most frequently observed in natural conditions, potentially accounting for the kinetics of the splicing reaction and the ligated exon rate, which were similar to those for the wild-type sequence. In addition, sequence analysis on the products of the excision reaction for the 1883U and 1883A mutants showed lariat and circular products, including truncated domain 6 linked to 5′ exon/intron circles and circular products with an extra cytidine residue (Table 3). Interestingly, true circles were detected for the 1883U mutant.

Substitutions involving the γ–γ′ interaction resulted in a lower efficiency of the first step of splicing, regardless of the maintenance of the Watson–Crick interaction (Table 1). The second step of splicing was also impaired in all the mutants, but mutations resulting in disruption of the Watson–Crick interaction resulted in greater defects, with the production of larger amounts of some low-mobility forms (Fig. 5). Interestingly, sequencing of the rapidly migrating bands after gel extraction for the γ–γ′–CC mutant revealed the presence of true circles, circles carrying an extra C and atypical circles formed by a truncated domain 6 linked to the end of the 5′ exon, but no lariat product was detected (Table 3). The γ–γ′ mutants maintaining Watson–Crick pairings displayed a more moderate effect on the second splicing step and ligated exon rates, and only the γ–γ′–AU mutant, which is as frequent in natural conditions as the WT G(γ)C(γ′) (Dai et al. 2003; Candales et al. 2012) displayed lower levels of accumulation of atypical low-mobility forms (Fig. 5), and an enrichment in the lariat product (Table 3).

Finally, substitutions involving the IBS3–EBS3 interaction did not impair the first step of splicing. Moreover, only the EBS3–IBS3–GG and EBS3–IBS3–CC mutants displayed defects in the second step of splicing, resulting in lower ligated exon rates (Tables 1, 2). The EBS3–IBS3–CG and EBS3–IBS3–UA mutants, for which Watson–Crick base-pairing was maintained, and intriguingly, also the EBS3–IBS3–AA mutant, for which Watson–Crick interaction was not conserved, displayed no defects in the second splicing step (Tables 1, 2). The EBS3–IBS3–CG and EBS3–IBS3–AA mutants resulted in an enrichment in lariat forms, whereas the EBS3–IBS3–UA mutant yielded a mixture of lariat and circular splicing products.

Our results suggest that the run of four cytidine residues in this intron plays an important role in regulating the fidelity of the RmInt1 splicing reaction and the formation of circular molecules, reflecting the high degree of complexity of these long-range interactions.

## DISCUSSION

Group II intron splicing is a complex process involving a large number of tertiary intramolecular interactions, with fine structural rearrangements of the intron occurring between the first and the second splicing step reactions (Marcia and Pyle 2012; Marcia et al. 2013). We showed that the removal of a sequence predicted to interfere with the splicing reaction from the 3′ exon led to an increase in splicing product fidelity. However, other atypical circular forms comigrating with the lariat intron were nevertheless observed. We hypothesize that the presence of a run of four cytidine residues in the 3′ SS might be responsible for the activation of cryptic sites and the formation of atypical circular forms. We observed that certain mutations affecting the EBS3–IBS3 interaction led to an increase in the rate of lariat formation (fidelity) while maintaining the ligated exon rate (efficiency). Based on these observations, we suggest that RmInt1 has evolved to preserve a splicing reaction with an intrinsic low efficiency and fidelity. We interpret this as an adaption favoring survival within the bacterial genome in which the RmInt1 lives, thus preventing the harmful insertion of the intron into essential sequences during its dispersion within the host genome.

### A predicted alternative IBS1 sequence in the 3′ exon affects splicing specificity

The RmInt1 intron has been shown to generate unusual splicing side products in vitro (Costa et al. 2006a). A sequence present within the 3′ exon has been predicted to match the EBS1 site better than the authentic IBS1 sequence at the 3′ end of the 5′ exon, leading to hydrolysis at positions +10 or +11 of the 3′ exon (Costa et al. 2006a). We deleted this sequence from the 3′ exon and showed that this deletion greatly decreased the number of atypical splicing forms generated (Fig. 3B). Interestingly, the rate of reaction was not affected, as expected given that the competing pathway occurs after the completion of the first splicing step (Costa et al. 2006a). However, the amplitude of the reaction was almost 20% higher in the absence of IBS1* sequence. This suggests that the IBS1* sequence interacting with the EBS1 led to the misfolding of a fraction of the population of precursor molecules, which precluded them from reacting. Alternative splice site sequences have been reported for several group II introns (Jacquier and Jacquesson-Breuleux 1991; Tourasse et al. 2005; Toor et al. 2006; Nagy et al. 2013). These regions generally include modified IBS1 motifs that translocate the intron interaction, generating alternative excision products, as observed for RmInt1 (Costa et al. 2006a). RmInt1 splicing in vivo is known to be assisted by the intron-encoded protein (IEP), which may even modulate the ratio of lariat and circular products (Molina-Sánchez et al. 2006). We therefore cannot rule out the possibility that the IEP impedes these alternative base-pairings in vivo or even that exon shuffling may be of biological relevance.

### Mutations of the run of cytidines at the 3′ splice site favor the production of lariat introns over unproductive circular forms

An analysis of the low-mobility splicing products of the ΔORF-WT2 transcript lacking the IBS1* sequence revealed the presence of unexpected circular forms comigrating with lariat products. These circular products included circular introns containing an extra residue belonging to the first nucleotide of the 3′ exon and intron circles consisting of an intron form with a truncated domain 6 linked to either the end of the 5′ exon or the beginning of the intron. It has been suggested that circles consisting of an intron with a truncated domain 6 linked to the end of the 5′ exon may be generated by transesterification between the 3′ OH group of the 3′ end of the truncated intron and the precursor triphosphate 5′ end (Costa et al. 2006a). RmInt1 domain 6 was generally truncated at the level of the η′ motif, consisting of a GAAA tetraloop. We hypothesize that the sequence 5′-GGUGAAA-3′ located at the distal portion of domain 6 can be recognized by EBS1, which is partially complementary to the above sequence, thus leading to hydrolysis and intron truncation at this position (Fig. 4B). However, the mechanism underlying the formation of intron forms with a truncated domain 6 linked to the 5′ end of the intron remains to be elucidated. We observed no true circles for the wild-type intron, although these forms have been characterized for other group II introns in vitro (Murray et al. 2001; Nagy et al. 2013) and for RmInt1 intron in vivo (Molina-Sanchez et al. 2006). It has been suggested that circles are generated by two successive transesterification reactions: A free 5′ exon attacks the 3′ SS, generating a 5′ exon–intron intermediate, and the 2′ OH of the terminal cytidine then reacts at the 5′ splice site (5′ SS) (Murray et al. 2001). This *trans*-splicing mechanism produces a circular intron together with ligated exons and a free 5′ exon, which can reinitiate the mechanism in another precursor molecule. We hypothesize that circles containing an extra nucleotide may form due to a lack of fidelity at the 3′ SS, the residues of which have been shown to affect the second step of splicing (Jacquier and Michel 1990; Chanfreau and Jacquier 1993; Costa et al. 2000). The RmInt1 intron 3′ SS consists of a run of four cytidine residues. We therefore created a series of mutant introns to investigate the role of this run of cytidine residues in the fidelity of RmInt1 excision at the 3′ SS. These mutants were designed to mimic the residue composition of the 3′ SS of high-fidelity introns, such as the ai5γ intron of the yeast mitochondrial *oxi* 3 gene (Arnberg et al. 1980), the Pl.LSU/2 intron from mitochondria of the alga *Pylaiella littoralis* (Costa et al. 1997) and the bacterial intron *O.i.*I1 from *Oceanobacillus iheyensis* (Takaki et al. 2004). We examined the efficiency of the first and second splicing steps by carrying out a kinetic analysis of the rates and products of the reaction and assessing its fidelity by sequencing the splicing products of these mutants (Tables 1–3; Fig. 5). We showed that mutations affecting the γ–γ′ interaction (γ–γ′–AU) increased the fidelity of the reaction, as shown by the smaller number of atypical splicing products and an enrichment in lariat forms. However, the efficiency of both the first and second steps of the reaction was diminished, resulting in a low rate of exon ligation. According to the available crystal structures (Marcia and Pyle 2012; Marcia et al. 2013), the γ (452) position is not located close to the catalytic center. However, modifications to the γ′ (1884) position, which is adjacent to the scissile phosphate, may interfere in the general architecture of the intron, affecting the availability of the catalytic elements and, thus, the efficiency of the reaction. Furthermore, mutations affecting the EBS3–IBS3 interaction increased both the efficiency and fidelity of the RmInt1 intron splicing reaction. EBS3–IBS3–CG and EBS3–IBS3–AA mutants displayed no impairment of reaction efficiency, and both produced a higher proportion of lariat forms. The EBS3–IBS3–AA mutant may restore the WT phenotype by means of a non-Watson–Crick interaction between the two adenosine residues, a combination that has not been observed in natural conditions (Simon et al. 2008). According to the available group II intron structures (Marcia and Pyle 2012; Marcia et al. 2013), two adenosine residues in the EBS3 and IBS3 positions would be able to engage in *cis*-Watson–Crick/Watson–Crick or *cis*-Watson–Crick/sugar-edge interactions (Leontis and Westhof 2001; Leontis et al. 2002; Nasalean et al. 2009).

The increase in the proportion of lariat forms was accompanied by high rates of exon ligation for the EBS3–IBS3–CG and EBS3–IBS3–AA mutant introns. In contrast, the γ–γ′–CC mutant yielded only circular forms and hardly any ligated exons. The γ–γ′–CC mutant has been reported to produce lariat-derived products in vivo (Molina Sanchez et al. 2011), but the methods used in those assays did not distinguish between intermediate forms linked to the 3′exon and fully excised lariat intron. In addition, the γ–γ′–AA mutant, which also displayed a strong defect of exon ligation, gave rise to far fewer excised products of the size of a lariat/circular intron (Fig. 5). Then, we observe a correlation between lariat intron formation and ligated exons. We suggest that atypical circle formation would be associated to higher rates of free exons, since we were not able to detect incorrect ligated exons.

### The RmInt1 intron uses various mechanisms to control the dispersion of harmful genetic elements in its bacterial host

In natural conditions, the RmInt1 intron is found in the region encoding the transposase of an IS*Rm2011-2* insertion sequence (Selbitschka et al. 1995; Martinez-Abarca et al. 1998). Several stop codons in the sequence of the intron result in a premature end to the translation of the transposase gene, rendering intron splicing essential for the mobility of the insertion sequence in the host genome (Martinez-Abarca et al. 1998). The location of the RmInt1 intron in a non-essential gene contrasts with the observed situation for mitochondrial group II introns, most of which are inserted into indispensable genes and require efficient splicing to ensure the survival of their hosts (Arnberg et al. 1980; Schmidt et al. 1990; Fontaine et al. 1995; Simon et al. 2008). With the lack of pressure for their efficient splicing, it has been suggested that RmInt1 and other bacterial group II introns behave essentially as retroelements rather than introns (Dai and Zimmerly 2002; Chillón et al. 2011). This study revealed some of the mechanisms used by RmInt1 to regulate its splicing efficiency and fidelity and, hence, the expression of the insertion sequence it interrupts. The first mechanism by which RmInt1 controls splicing concerns the ligation of exons by its transposase. It has already been demonstrated in vivo that the efficiency of exon ligation by the RmInt1 intron is extremely low, with <1% of exons correctly spliced (Chillón et al. 2011). It has already been suggested, and we confirm here, that the presence of an alternative IBS1 sequence in the 3′ exon leads to hydrolytic cleavage at position +10 or +11 of the 3′ exon, leading to the irreversible loss of a fraction of functional transposase mRNA molecules (Costa et al. 2006a). Finally, we suggest here that the existence of a run of four cytidine residues spanning the 3′ SS, and probably leading to an increase in the generation of unproductive intron circles, also helps to reduce expression of the transposase. Indeed, similar runs of cytidine residues have been detected in other bacterial group II introns of class D, such as the Sr.md.I1 intron in *Sinorhizobium medicae*, the Pr.ae.I3 intron in *Prosthecochloris aestuarii*, the Pr.vi.I1 intron in *Prosthecochloris vibrioformis* and the Zu.pr.I1 intron in *Zunongwangia profunda* (Candales et al. 2012). Interestingly, all these introns interrupt non-essential genes encoding transposases and integrases, for example, suggesting that the same mechanism may operate in other bacterial group II introns.

## CONCLUSION

The experiments reported here highlight various mechanisms by which the RmInt1 group II intron may fine-tune its splicing efficiency and fidelity as a means of controlling the dispersion of mobile genetic elements in the host genome. We confirmed that the predicted alternative IBS1* sequence in the 3′ exon was able to decrease the splicing fidelity of the RmInt1 intron. We also suggest that a run of cytidine residues in the 3′ SS may be responsible for the lack of fidelity in recognition of the 3′ SS. This may prevent the intron from adopting the three-dimensional conformation necessary for correct 3′ splice site selection and exon ligation. We showed that mutations affecting the EBS3–IBS3 interaction improved efficiency and the fidelity at the 3′ SS. Together, these mechanisms ensure low levels of expression of the insertion sequence interrupted by RmInt1 in its natural context, helping to ensure the survival of the bacterial host.

## MATERIALS AND METHODS

### Plasmid constructs

ΔORF-WT2 was derived from ΔORF-WT (previously named as Δ15 or pLM1) (Costa et al. 2006a). Both constructs are derived from the pUC19 plasmid. The 3′ end of ΔORF-WT consists of 146 bp of the RmInt1 3′ exon, corresponding to the sequence of IS*Rm2011-2*, resulting in a 911-nt transcript. We created ΔORF-WT2, by using the 5Q_CAT (5′-TGTACCTATAACCAGACCGTTCAG) and 27PR_lacZtranscript (5′-GGAGATCTCATATGACTGTTGGGAAGGGCGA) primers to amplify a 1009-bp fragment of pICG-ΔORF (Chillón et al. 2011), which was then inserted between the ClaI and BamHI sites of pLM1. Mutant ΔORF- WT2-derivative plasmids were generated by cloning a ClaI–BglII fragment containing the mutated position. Site-directed mutagenesis was performed as described by Higuchi et al. (1988) (Supplemental Table S1). The sequences of the mutant constructs were checked by sequencing. Thus, the 3′ region of ΔORF-WT2 consists of 5 bp of the RmInt1 3′ exon from IS*Rm2011-2* and 145 bp of the *E. coli lacZ* gene (positions 42–169) flanked by restriction sites, making it possible to produce two transcripts of 775 and 908 nt, respectively.

### In vitro transcription and purification

The ΔORF-WT template was generated by BamHI digestion. ΔORF-WT2 templates were generated by NdeI or BamHI digestion to produce the long and short transcripts, respectively. Transcription was performed with 2–4 units of T7 RNA polymerase, 4 µg of linearized plasmid, 1× transcription buffer [10× transcription buffer: 150 mM MgCl_2_, 400 mM Tris–HCl, pH 7.5, 20 mM Spermidine, and 50 mM DTT], 0.96 mM ATP, 0.96 mM CTP, 0.96 mM UTP, 0.064 mM GTP, 50 µCi [α-^32^P]GTP (3000 Ci/mmol; 10 mCi/mL; PerkinElmer), 10 mM DTT and 80 units of RNAseOUT (Invitrogen), in a final volume of 50 µL (Pyle and Green 1994). Transcription was stopped after 1.5 h at 37°C, by adding an equal volume of gel loading dye (10 M urea, 0.1% [w/v] xylene cyanol and bromophenol blue dyes, 40 mM Tris [pH 7.5], 8.3% [w/v] sucrose, and 0.83 mM EDTA) and running directly on a 5% (w/v) denaturing (7 M urea) polyacrylamide gel. After migration, the transcripts were visualized by autoradiography (10-min exposure), excised from the gel, and eluted by incubation for 3 h in elution buffer (0.3 M NaCl, 10 mM MOPS pH 6, 1 mM EDTA). RNA was then isolated by ethanol precipitation of the eluate (three volumes of 100% ethanol and 40 µg of glycogen [Roche]). The RNA was dried and dissolved in 30 µL of RNA storage buffer (10 mM MOPS pH 6, 1 mM EDTA).

### Self-splicing assays

The precursor RNA was incubated in 80 mM MOPS buffer pH 7.5 at 95°C for 1 min. The RNA sample was then allowed to cool to reaction temperature before being combined with 500 mM (NH_4_)_2_SO_4_ (final concentration). Reactions were initiated by adding 100 mM MgCl_2_ (final concentration) and were incubated at 50°C. For time-course analyses, aliquots (1–2 µL) were removed at the indicated time points, added to quenching buffer (1.8% sucrose, 1× TBE [Tris–borate EDTA, pH 8.3], 0.018% xylene cyanol dye, 36% [v/v] formamide, and 25 mM EDTA) and placed on ice to stop the reaction. Samples were then loaded onto a 5% denaturing polyacrylamide gel and the products were resolved by electrophoresis. The gels were dried and placed against phosphorus plates (Imaging Plate 2040, Fujifilm). Images were acquired after 24–48 h of exposure, with the Personal Molecular Imager FX (Bio-Rad) laser scanning system, and quantified with Quantity One software (Bio-Rad).

### Identification of in vitro splicing products

Total splicing reactions incubated for 2 h were subjected to electrophoresis in denaturing gels and low-mobility bands were purified. Reverse transcription and PCR were performed to detect the formation of lariat and circle forms, as previously described (Molina-Sanchez et al. 2006). The PCR products were inserted into the pGEM-T Easy Vector (Promega) and after transformation of *E. coli* cells the isolated clones were amplified by colony PCR and sequenced. For the identification of ligated exons, an unfractionated splicing reaction (2 h) mixture was subjected to reverse transcription with the 6QR_LACZ (5′-GATGTGCTGCAAGGCGATT) primer, followed by PCR amplification with this primer and 30P_5exonpLM (5′-GGGAATTTTCATCGATGAGA). The resulting PCR products were cloned and sequenced, as described above.

### Kinetic analysis of time courses

Individual bands on autoradiographs were quantified with Quantity One software (Bio-Rad). Counts for a nearby position in the same lane at which there was no band present were subtracted, to provide an internal measurement of background RNA degradation at each time point. The relative fractions of precursor and products were calculated and corrected for the guanosine content of each species (Daniels et al. 1996). We then used Prism 6 (GraphPad Software, Inc.) to fit a single exponential decay equation with endpoint correction to the data for wild-type transcript reactivity. This equation took the form (*y*_0_ − *y*_final_) × *e*[−*k*_obs_(*t*)] + *y*_final_, where *y*_0_ is the starting point, *y*_final_ is the endpoint, and *k*_obs_ is the reaction rate. Computer simulations were performed with KinTek Explorer (KinTek Corporation), for the fitting of the data.

### SYBR Green quantitative PCR

We amplified and quantified ligated exons, using the 30P_5exonpLM and 6QR_LACZ primers, which span the splicing junction. The RT-qPCR experiments were conducted with a LightCycler 480 System (Roche). Each reaction was run in triplicate and contained l µg of cDNA template, 1 pmol of each oligonucleotide primer, 2× Master Mix I (Roche), and water, added to a final volume of 10 µL. The PCR cycling conditions were a 5-min hot start at 95°C, followed by 45 cycles of denaturation at 95°C for 10 sec, annealing at 63°C for 2 sec, and extension at 72°C for 2 sec. Melting curve analysis was performed for all PCR runs, to check the identity of the PCR product and the specificity of the primers. We assessed mRNA levels by relative quantification, according to the 2^−ΔΔCT^ method (Livak and Schmittgen 2001). The normalized expression ratio was defined as 2^−ΔΔCT^, where ΔΔ*C*_T_ = Δ*C*_T_ (calibrator or WT) − Δ*C*_T_ (test or mutant), and is expressed as a percentage of the value for the WT construct.

## SUPPLEMENTAL MATERIAL

Supplemental material is available for this article.

## Supplementary Material

Supplemental Material
