# Supplementary material for: In vitro characterization of the splicing efficiency and fidelity of the RmInt1 group II intron as a means of controlling the dispersion of its host mobile element
Source: RNA. 2014 Dec;20(12):2000–10. doi: 10.1261/rna.047407.114 (PMC4238363; doi:10.1261/rna.047407.114)
Supplement: Supplemental Material [file supp_20_12_2000__index.html]

In vitro characterization of the splicing efficiency and fidelity of the RmInt1 group II intron as a means of controlling the dispersion of its host mobile element — In vitro characterization of the splicing efficiency and fidelity of the RmInt1 group II intron as a means of controlling the dispersion of its host mobile element — Supplemental Material 

# In vitro characterization of the splicing efficiency and fidelity of the RmInt1 group II intron as a means of controlling the dispersion of its host mobile element

## Supplemental Material

**Files in this Data Supplement:**

- Supp Table S1.docx
