# Supplementary material for: In vitro characterization of the splicing efficiency and fidelity of the RmInt1 group II intron as a means of controlling the dispersion of its host mobile element
Source: RNA. 2014 Dec;20(12):2000–10. doi: 10.1261/rna.047407.114 (PMC4238363; doi:10.1261/rna.047407.114)
Supplement: Supplemental Material [file supp_047407.114_TABLE_S1.docx]

| **TABLE S1.** Primers used for the site-directed mutagenesis of ΔORF-WT2 | | |  |
| --- | --- | --- | --- |
| **Mutant** | **PCR Primer^1^** | **Sequence** | **Reference** |
| 1883A | 5Q_CAT  22PR_C1883A | TGTACCTATAACCAGACCGTTCAG  CCCCTAGGCCAGGGTTGAGTAGG | (Chillon *et al.* 2011)  This work |
| 1883G | 5Q_CAT  23PR_C1883G | TGTACCTATAACCAGACCGTTCAG  CCCCTAGGCCAGGGCTGAGTAGG | (Chillon *et al.* 2011)  This work |
| 1883U | 5Q_CAT  24PR_C1883T | TGTACCTATAACCAGACCGTTCAG  CCCCTAGGCCAGGGATGAGTAGG | (Chillon *et al*. 2011)  This work |
| γ-γ’-CC | **5Q_CAT**  γC-rv  γC-fw  **6QR_LACZ** | TGTACCTATAACCAGACCGTTCAG TACGGGGCGATCGGCCCCAGTCTCCTGCATC  GACTGGGGCCGATCGCCCCGTATGAGCGTCGAGG  GATGTGCTGCAAGGCGATT | (Chillon *et al*. 2011)  (Molina Sanchez *et al.* 2011)  (Molina Sanchez *et al.* 2011)  (Chillon *et al*. 2011) |
| γ-γ’-GG | 5Q_CAT  γ’G | TGTACCTATAACCAGACCGTTCAG  CACCTAGGCCAGGCGTGAGTAGG | (Chillon *et al.* 2011)  (Molina Sanchez *et al*. 2011) |
| γ-γ’-CG | **5Q_CAT**  γC-rv  γC-fw  **γ’G** | TGTACCTATAACCAGACCGTTCAG  TACGGGGCGATCGGCCCCAGTCTCCTGCATC GACTGGGGCCGATCGCCCCGTATGAGCGTCGAGG  CACCTAGGCCAGGCGTGAGTAGG | (Chillon *et al.* 2011)  (Molina Sanchez *et al.* 2011)  (Molina Sanchez *et al.* 2011)  (Molina Sanchez *et al.* 2011) |
| γ-γ’-AU | **5Q_CAT**  7OR_G452A  7O_G452A  **29PR_C1884U** | TGTACCTATAACCAGACCGTTCAG TACGGGGCGATCTGCCCCAGTCTCCTGCATC  GACTGGGGCAGATCGCCCCGTATGAGCGTCGAGG  CCCCTAGGCCAGGAGTGAGTAGG | (Chillon *et al.* 2011)  This work  This work  This work |
| γ-γ’-AA | **5Q_CAT**  7OR_G452A  7O_G452A  **30PR_C1884A** | TGTACCTATAACCAGACCGTTCAG TACGGGGCGATCTGCCCCAGTCTCCTGCATC  GACTGGGGCAGATCGCCCCGTATGAGCGTCGAGG  CCCCTAGGCCAGGTGTGAGTAGG | (Chillon *et al*. 2011)  This work  This work  This work |
| EBS3-IBS3-CC^2^ | 5Q_CAT  6QR_LACZ | TGTACCTATAACCAGACCGTTCAG  GATGTGCTGCAAGGCGATT | (Chillon *et al*. 2011)  (Chillon *et al*. 2011) |
| EBS3-IBS3-GG | **5Q_CAT**  32PR_C+1G | TGTACCTATAACCAGACCGTTCAG CCCCTAGGCCAGCGGTGAGTAGG | (Chillon *et al*. 2011)  This work |
| EBS3-IBS3-CG | **5Q_CAT**  10OR_GEBS3C 10O_GEBS3C  **32PR_C+1G** | TGTACCTATAACCAGACCGTTCAG CACGCCCCCTGTACGCCGGTCGCCGCCC  GACCGGCGTACAGGGGGCGTGAGTTGGACATAGG CCCCTAGGCCAGCGGTGAGTAGG | (Chillon *et al*. 2011)  This work  This work  This work |
| EBS3-IBS3-UA | **5Q_CAT**  8OR_GEBS3U  8O_GEBS3U  **31PR_C+1A** | TGTACCTATAACCAGACCGTTCAG CACGCCCCCTATACGCCGGTCGCCGCCC  GACCGGCGTATAGGGGGCGTGAGTTGGACATAGG  CCCCTAGGCCAGTGGTGAGTAGG | (Chillon *et al.* 2011)  This work  This work  This work |
| EBS3-IBS3-AA | **5Q_CAT**  9OR_GEBS3A  9O_GEBS3A **31PR_C+1A** | TGTACCTATAACCAGACCGTTCAG CACGCCCCCTTTACGCCGGTCGCCGCCC  GACCGGCGTAAAGGGGGCGTGAGTTGGACATAGG CCCCTAGGCCAGTGGTGAGTAGG | (Chillon *et al*. 2011)  This work  This work  This work |
| ^1^ Bold letters indicate the outer primers used in secondary PCR.  ^2^ The primers indicated were used to amplify this mutant, which was previously described by Molina-Sanchez *et al.* (Molina Sanchez *et al*. 2011), with the appropriate flanking restriction sites. | | | |
